# Telomere G-tail Length is a Promising Biomarker Related to White Matter Lesions and Endothelial Dysfunction in Patients With Cardiovascular Risk: A Cross-sectional Study^☆^

**DOI:** 10.1016/j.ebiom.2015.05.025

**Published:** 2015-05-30

**Authors:** Tomohisa Nezu, Naohisa Hosomi, Tetsuya Takahashi, Kumiko Anno, Shiro Aoki, Akira Shimamoto, Hirofumi Maruyama, Tomonori Hayashi, Masayasu Matsumoto, Hidetoshi Tahara

**Affiliations:** a Department of Clinical Neuroscience and Therapeutics, Hiroshima University Graduate School of Biomedical and Health Sciences, Hiroshima, Japan; b Department of Cellular and Molecular Biology, Institute of Biomedical & Health Sciences, Hiroshima University, Hiroshima, Japan; c Department of Radiobiology/Molecular Epidemiology, Radiation Effects Research Foundation, Hiroshima, Japan

**Keywords:** Endothelial dysfunction, Telomere lengths, Telomere G-tail lengths, White matter changes

## Abstract

**Background:**

The telomeric 3′-overhang (G-tail) length is essential for the biological effects of telomere dysfunction in vitro, but the association of length with aging and cardiovascular risk is unclear in humans. We investigated the association between the telomere G-tail length of leukocytes and cardiovascular risk, age-related white matter changes (ARWMCs), and endothelial function.

**Methods:**

Patients with a history of cerebrovascular disease and comorbidity were enrolled (n = 102; 69 males and 33 females, 70.1 ± 9.2 years). Total telomere and telomere G-tail lengths were measured using a hybridization protection assay. Endothelial function was evaluated by ultrasound assessment of brachial flow-mediated dilation (FMD).

**Findings:**

Shortened telomere G-tail length was associated with age and Framingham risk score (P = 0.018 and P = 0.012). In addition, telomere G-tail length was positively correlated with FMD values (P = 0.031) and negatively with the severity of ARWMCs (P = 0.002). On multivariate regression analysis, telomere G-tail length was independently associated with FMD values (P = 0.022) and the severity of ARWMCs (P = 0.033), whereas total telomere length was not associated with these indicators.

**Interpretation:**

Telomere G-tail length is associated with age and vascular risk factors, and might be superior to total telomere length as a marker of endothelial dysfunction and ARWMC severity.

## Highlights

• Telomere G-tail length was measured using a hybridization protection assay in patients with vascular risk factors.
• Telomere G-tail length was independently related to endothelial function and age-related white matter changes.
• Telomere G-tail length might be a promising biomarker of vascular damage.

## Graphical abstract

**Figure:**
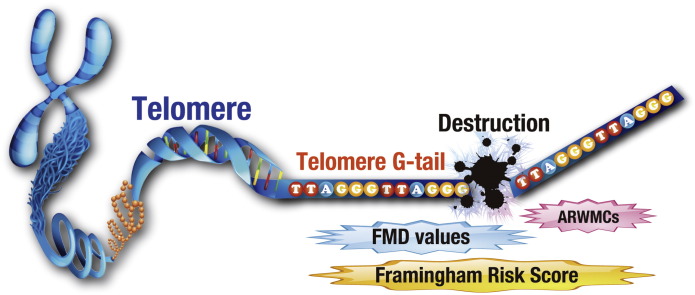

## 1 Introduction

Telomeres are the structures which cap each end of a chromatid, at the extreme end of chromosomal deoxyribonucleic acid (DNA). Telomeres are composed of a variable number of tandem repeats of the sequence TTAGGG that extend over several thousand base pairs (Blackburn, 2001). The protected state of telomeres is necessary to safeguard the integrity of genomic material. Telomere length is considered a biomarker of human aging, namely an indicator of oxidative stress and cellular senescence (von Zglinicki and Martin-Ruiz, 2005). Additionally, telomere shortening in leukocytes is correlated with atherosclerosis and cardiovascular aging (De Meyer et al., 2011, Butt et al., 2010, Khan et al., 2012). Telomeric DNA is shielded by telomere binding-specific proteins, such as the ‘shelterin complex’, which bind to and protect the terminal loop (t-loop) of telomeres (Bailey et al., 2001, de Lange, 2005). Telomeres are composed of double-stranded repeated DNA with terminal 3′ single-stranded G-rich overhangs called telomere G-tails. The telomere G-tail folds back to form a t-loop, which prevents the end of the telomere from being recognized as a damaged, broken end. Telomere G-tails are thus key structures that protect telomere DNA from DNA damage. Indeed, the telomere G-tail is thought to be a more important factor in chromosome maintenance than total telomere length (Tsai et al., 2007, Anno et al., 2007). However, the association of telomere G-tail length with various cardiovascular risk factors in humans has not been clarified. To our knowledge, only one report has shown an association between the telomere G-tail and cardiovascular events in hemodialysis patients (Hirashio et al., 2014).

Endothelial dysfunction plays a critical role in the development of atherosclerosis. Impairment of endothelial function is an important first step in the pathogenesis of atherosclerosis, and is associated with an insufficient endothelial repair mechanism for vascular injury (Deanfield et al., 2007). Ultrasound assessment of brachial artery flow-mediated dilation (FMD), a useful noninvasive method, has been used as an index of endothelium-dependent vasodilation. Several studies have reported that FMD is impaired in patients with coronary disease and cardiovascular risk factors. In addition, impaired FMD is associated with the severity of age-related white matter changes (ARWMCs) (Hoth et al., 2007). However, the association of telomere G-tail length with endothelial dysfunction and ARWMCs remains unclear.

Here, we investigated associations among telomere G-tail length in leukocytes, aging, vascular risk factors and endothelial dysfunction in patients with chronic cerebrovascular disease and comorbidities.

## 2 Methods

### 2.1 Study Participants

The study was conducted under a single-center, hospital-based prospective design. The study protocol was governed by the guidelines of the Japanese government, based on the Helsinki Declaration as revised in 1983. The study was approved by the Institutional Research and Ethics Committee of our hospital. All patients provided written informed consent to participate. Patients treated for cerebrovascular disease (at least 6 months after stroke onset) or atypical neurological problems (e.g., dizziness, headache, numbness and other symptoms) at Hiroshima University Hospital between November 2012 and April 2014 were enrolled. All patients underwent magnetic resonance imaging (MRI) and FMD studies, with the MRI study performed within 3 months before or after the FMD study. Baseline clinical characteristics were recorded, including age, sex, hypertension, diabetes mellitus, dyslipidemia, atrial fibrillation, renal dysfunction, history of stroke (ischemic or hemorrhagic), coronary artery disease and current smoking status. In addition to obtaining a medical history, relevant risk factors were identified from the self-reported medical history or were inferred from medications prescribed by the primary physician. The criteria for hypertension, diabetes mellitus and dyslipidemia were as previously defined (Hosomi et al., 2012). Framingham risk score was calculated according to the presence of the following cardiovascular risk factors: age, sex, total cholesterol level, high-density lipoprotein cholesterol level, systolic blood pressure (treated or not treated), diabetes mellitus and smoking status (D'Agostino et al., 2008). Renal function was calculated using the estimated glomerular filtration rate (eGFR) with a revised equation for the Japanese population, as follows: eGFR (ml/min/1.73 m^2^) = 194 × (serum creatinine)^− 1.094^ × (age)^− 0.287^ × 0.739 (for women) (Matsuo et al., 2009). Renal dysfunction was defined as an eGFR < 60 ml/min/1.73 m^2^. Serum high-sensitivity C-reactive protein (hs-CRP) was measured using a CRP-Latex kit (Nissui Pharmaceutical Co. Ltd., Tokyo, Japan) according to the manufacturer's instructions. The FMD study and blood sample collection were performed in the morning on the same day. To enroll control subjects, blood samples were obtained from 493 volunteers. From these, we selected control subjects who were matched with our study participants for age and sex, and finally assessed 102 samples for telomere G-tail and total telomere lengths as control subjects.

### 2.2 FMD and Nitroglycerin-mediated Vasodilation (NMD)

The patients were requested to abstain from alcohol, smoking and caffeine on the day of the FMD examination. The FMD examination was conducted during the fasting state in the morning, and only drinking water was given to the patients. Most medications taken by the patients were withheld, and only those deemed necessary (such as antithrombotic therapies) were administered, at the discretion of the attending physician. The FMD was measured with a high-resolution linear artery transducer that was coupled to computer-assisted analysis software (UNEXEF38G, UNEX Co., Nagoya, Japan) as detailed previously (Maruhashi et al., 2012, Iwamoto et al., 2012). Briefly, the FMD protocol was as follows: a blood pressure cuff was placed around the forearm of the patient. The brachial artery was scanned longitudinally 5–10 cm above the elbow with a special probe holder (UNEX Co.) to ensure the consistency of the B-mode image. The diameter of the artery was automatically tracked, and the waveform of diameter changes over the cardiac cycle was displayed in real time using the FMD mode of the tracking system. A baseline longitudinal image of the artery was acquired for 30 s, and the blood pressure cuff was inflated to 50 mm Hg above systolic pressure for 5 min. Pulsed Doppler velocity signals were obtained for 20 s at baseline and for 10 s immediately after cuff deflation. Changes in the diameter of the brachial artery were immediately expressed as the percent change relative to the vessel diameter before cuff inflation. The FMD was automatically calculated as the percent change in peak vessel diameter from the baseline value, and %FMD (peak diameter − baseline diameter / baseline diameter) was used for analysis. The nitroglycerin response was used to measure endothelium-independent vasodilation. After acquiring a baseline resting image for 30 s, a 75-μg tablet of nitroglycerin was administered sublingually, and images of the artery were recorded continuously for 8 min. The NMD was automatically calculated as the percent change in peak vessel diameter from the baseline value, and %NMD (peak diameter − baseline diameter / baseline diameter) was used for analysis. A single investigator (T.N.) who was unaware of the clinical details of the patient performed the FMD and NMD evaluations for all patients. The intra-observer coefficients of variation were 1.2% for baseline brachial artery diameter among all patients and 11.1% for FMD in 18 randomly selected patients.

### 2.3 Magnetic Resonance Imaging

MRI was performed with a 1.5T scanner (Signa, GE Medical Systems, Fairfield, CT, USA or Magneton Symphony Advanced or Avanto, Siemens Medical Systems, Erlangen, Germany) or 3.0T scanner (Signa, GE Medical Systems). The imaging protocol consisted of a T1-weighted spin-echo and a T2-weighted spin-echo and fluid-attenuated inversion recovery (FLAIR). A single stroke neurologist (T.N.) who was unaware of the clinical details of the patients graded the severity of the ARWMCs. The severity of ARWMCs was rated visually from the FLAIR images using the Fazekas scale (grade 0, no lesions; grade 1, punctate lesions; grade 2, early confluent lesions; and grade 3, confluent lesions) (Fazekas et al., 1987). Deep white matter changes (frontal, temporal, parietal, occipital) were assessed using the Scheltens scale score (score of 0 to 6: 0, absent; 1, < 3 mm in ≤ 5 lesions; 2, < 3 mm in ≥ 6 lesions; 3, 4–10 mm in ≤ 5 lesions; 4, 4–10 mm in ≥ 6 lesions; 5, ≥ 11 mm; and 6, confluent), giving a possible range of 0–24. Periventricular white matter changes (frontal caps, lateral bands, occipital caps) (range, 0–6) were assigned scores of 0 to 2 (0, absent; 1, < 5 mm; and 2, 5–10 mm) (Scheltens et al., 1993, Miwa et al., 2014). The sum of these ratings was used as the Scheltens rating score (range, 0–30). The Scheltens rating score was positively correlated with the Fazekas rating scores (ρ 0.849, P < 0.001).

### 2.4 Measurement of Leukocyte Total Telomere Length and Telomere G-tail Length

Peripheral blood samples were collected after FMD examination in each patient. Total telomere length was measured using telomere hybridization protection assay (HPA) methods (Hirose et al., 1997), and telomere G-tail length was measured using a telomere G-tail HPA (Tahara et al., 2005). Procedures are described in detail elsewhere (Anno et al., 2007, Tahara et al., 2005). Briefly, genomic DNA was isolated from the peripheral blood by the phenol–chloroform extraction method. The amount of DNA used in each assay was measured before assay using a Nanophotometer Pearl (Implen GmbH, Munich, Germany) with an AE-labeled telomere probe (Fujirebio Inc., Tokyo, Japan). G-tail telomere length was measured using 1 μg of non-denatured genomic DNA, and total telomere length was measured using 0.2 μg of denatured genomic DNA using a 96-plate formatted automated machine, JANUS® Automated Workstation, combined with an EnVision® Multilabel Plate Reader (PerkinElmer, Massachusetts U.S.A.). All samples were assessed in triplicate, with genomic DNA of HeLa cells used as control to correct for inter-assay variability. The average coefficient of variance in all samples was 4.8% for telomere G-tail length and 4.8% for total telomere length, while the inter-assay coefficient of variance was 6.2% for telomere G-tail length and 6.1% for total telomere length.

### 2.5 Statistical Analysis

Statistical analysis was performed using JMP 10.0 statistical software (SAS Institute Inc., USA). The data are expressed as the mean ± standard deviation (SD) or median (25th and 75th percentiles) for continuous variables and as frequency and percentage for discrete variables. The patients were divided into tertiles, with the numbers of subjects in order of telomere G-tail length or total telomere length (lowest tertile, middle tertile and highest tertile). The statistical significance of inter-group differences was assessed by the *χ^2^* test, unpaired *t*-test, Mann–Whitney *U* test and Kruskal–Wallis test, as appropriate. Relationships between telomere G-tail length (or total telomere length) and the other variables were examined by Spearman's correlation. In addition, relationships among FMD, ARWMCs and the other variables were also examined by Spearman's correlation. Indicators of the severity of endothelial dysfunction (FMD values) or ARWMCs were identified using multiple linear regression that included age, sex, body mass index, smoking history, hypertension, diabetes mellitus, dyslipidemia, atrial fibrillation, renal dysfunction, systolic blood pressure, diastolic blood pressure, history of stroke, history of coronary artery disease, laboratory findings and telomere G-tail length or total telomere length by a backward selection procedure using P > 0.10 for the likelihood ratio test as exclusion criterion. Statistical significance was established at P < 0.05.

## 3 Results

**Fig. 1:**
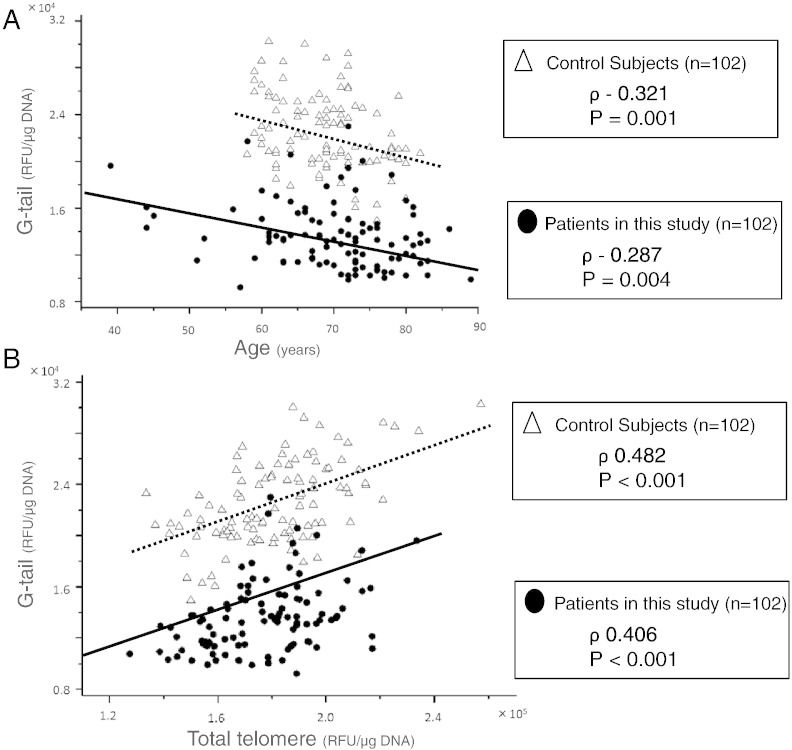
(A) Scatter plots representing the relationship between telomere G-tail length and age in patients and control subjects. (B) Scatter plots demonstrating the relationship between telomere G-tail length and total telomere lengths in patients and control subjects.

A total of 102 patients (69 males and 33 females, 70.1 ± 9.2 years) were registered in the study. Baseline clinical characteristics are presented in Table 1. Telomere G-tail length was negatively correlated with aging and positively correlated with total telomere length (ρ − 0.287, P = 0.004 and ρ 0.406, P < 0.001, Fig. 1). Neither telomere G-tail length nor total telomere length was associated with laboratory findings, including altered glucose levels, lipid levels, renal dysfunction or inflammation (Supplemental Table 1). Patients in this study had a shorter mean telomere G-tail length than control subjects (13653.0 ± 2787.4 RLU/μg DNA vs. 22504.9 ± 3249.1 RLU/μg DNA, P < 0.001), but did not significantly differ by total telomere length (Supplemental Table 2). Further, the groups significantly differed by distribution of the association between age and telomere G-tail length (Fig. 1A), and also significantly differed in the distribution of associations with total telomere length and telomere G-tail length (Fig. 1B).

### 3.1 Associations Between Telomere G-tail Length, Total Telomere Length and Vascular Risk Factors

**Fig. 2:**
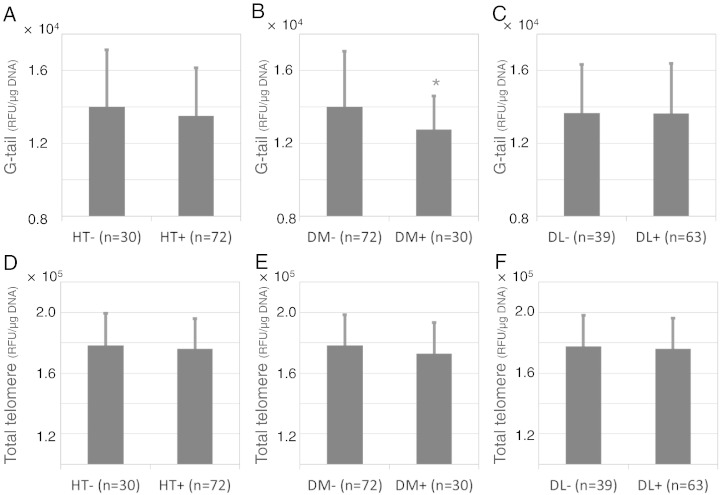
Relationships among telomere G-tail length, total telomere length and vascular risk factors. *P < 0.05. HT = hypertension; DM = diabetes mellitus; DL = dyslipidemia.

Associations between telomere G-tail length, total telomere length and traditional vascular risk factors (hypertension, diabetes mellitus and dyslipidemia) are presented in Fig. 2. Associations between total telomere length and vascular risk factors were not significantly different. In contrast, patients with diabetes mellitus had a shorter telomere G-tail length than those without (12755.4 ± 1831.2 RLU/μg DNA vs. 14027.0 ± 3025.8 RLU/μg DNA, P = 0.035). Patient characteristics according to telomere G-tail length tertile are presented in Table 2. A shorter telomere G-tail length (lowest tertile) was associated with age and a higher Framingham risk score (P = 0.018 and P = 0.012). Similarly, total telomere length (lowest tertile) was also associated with age and a higher Framingham risk score (P = 0.001 and P = 0.026) (Supplemental Table 3).

### 3.2 Association Between Telomere G-tail Length, Total Telomere Length and Endothelial Dysfunction

**Fig. 3:**
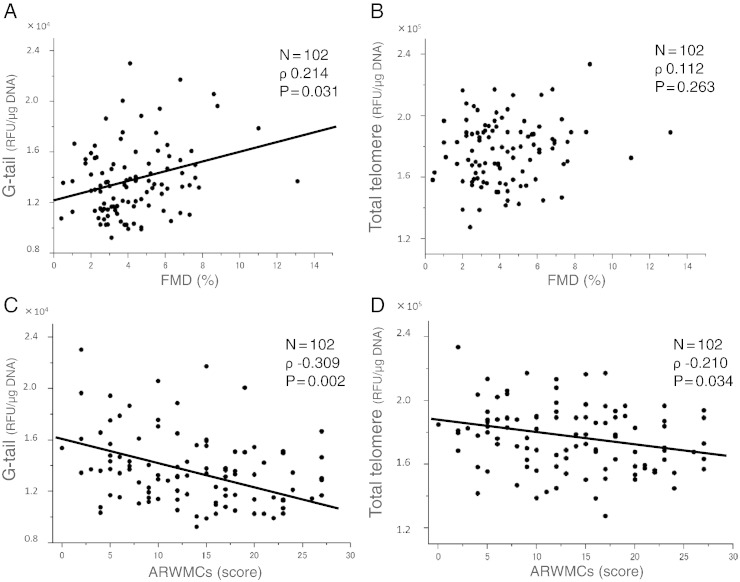
(A) Scatter plots demonstrating the relationship between telomere G-tail length and flow-mediated dilation (FMD). (B) Scatter plots demonstrating the relationship between total telomere length and FMD. (C) Scatter plots demonstrating the relationship between telomere G-tail length and age-related white matter changes (ARWMCs). (D) Scatter plots demonstrating the relationship between total telomere length and ARWMCs.

On univariate regression analysis, FMD was associated with age (ρ − 0.266, P = 0.007), male sex (ρ − 0.309, P = 0.002), BMI (ρ − 0.255, P = 0.010), hypertension (ρ − 0.305, P = 0.002), diabetes mellitus (ρ − 0.277, P = 0.005), renal dysfunction (ρ − 0.208, P = 0.036), high-density lipoprotein cholesterol level (ρ 0.254, P = 0.010), triglyceride level (ρ − 0.280, P = 0.004), eGFR (ρ 0.199, P = 0.045) and hs-CRP levels (ρ − 0.224, P = 0.024) (Table 3). Telomere G-tail length was positively correlated with FMD values (ρ 0.214, P = 0.031), whereas total telomere length was not significantly associated with FMD (ρ 0.112, P = 0.263) (Fig. 3A, B). Longer telomere G-tail length was independently associated with FMD value (standardized partial regression coefficient [β] 0.205; P = 0.022) on multivariate regression analysis for age, sex, comorbidity and other laboratory findings (Table 3). In contrast, total telomere length was not associated with FMD value on multivariate regression analysis using the backward selection procedure. In addition, total telomere length was not associated with FMD value after adjustment for age and sex (β 0.056; P = 0.574).

### 3.3 Associations Between Telomere G-tail Length, Total Telomere Length and Age-related White Matter Changes

Univariate regression analysis revealed that the severity of ARWMCs was associated with age (ρ 0.376, P < 0.001), hypertension (ρ 0.263, P = 0.008), HbA1c level (ρ 0.215, P = 0.030) and fasting blood glucose level (ρ 0.203, P = 0.041). Telomere G-tail length was negatively correlated with the severity of ARWMCs (ρ − 0.309, P = 0.002), and total telomere length was slightly associated with ARWMCs (ρ − 0.210, P = 0.034) (Fig. 3C, D). In multivariate regression analysis, shorter telomere G-tail length was independently associated with the severity of ARWMCs (β − 0.200; P = 0.033) (Table 4). In contrast, total telomere length was associated with the severity of ARWMCs on multivariate regression analysis using the backward selection procedure. In addition, total telomere length was not associated with ARWMCs after adjustment for age and sex (β − 0.071; P = 0.476).

## 4 Discussion

In this study, we demonstrated that the telomere G-tail length of leukocytes is significantly correlated with the endothelial function and severity of ARWMCs after adjustment for age, sex, traditional vascular risk factors and laboratory findings. In contrast, no significant association was seen between the total telomere length of leukocytes and these factors. Our study provides initial evidence for an association of telomere G-tail length with aging, endothelial function and ARWMCs in patients with vascular risk factors.

Several epidemiologic studies have demonstrated that shorter leukocyte telomere length is associated with atherosclerosis and cardiovascular risk factors (Brouilette et al., 2003, Demissie et al., 2006). Thus, the assessment of telomere length has been considered useful for the prediction of progressive atherosclerosis or cardiovascular events (De Meyer et al., 2011, Butt et al., 2010, Khan et al., 2012). Individuals with shorter telomeres may have an increased risk of early vascular cell aging and senescence, which causes progressive atherosclerosis. On the other hand, inflammation, oxidative stress, and insulin resistance, which are associated with cardiovascular disease, result in accelerated telomere attrition and shorter telomeres. Given that these indicators are also associated with endothelial dysfunction, we and others speculated that shorter telomeres might also be associated with endothelial dysfunction. Minamino et al. reported that endothelial senescence downstream of telomere function inhibition is related to endothelial dysfunction (increased intercellular adhesion molecule [ICAM]-1 expression and decreased endothelial nitric oxide synthase [eNOs] activity) in vitro (Minamino et al., 2002). Nakashima et al. assessed the cardiovascular damage (CVD) score (hypertension, dyslipidemia, diabetes, coronary artery disease, stroke and peripheral artery disease), endothelial function using FMD, and leukocyte telomere length by measuring mean telomere restriction fragment (TRF) length using the Southern blot technique in patients with cardiovascular risk factors (Nakashima et al., 2004). Although they found that CVD score was associated with FMD and leukocyte telomere length, it was unclear whether leukocyte telomere length was associated with FMD values. In our study, total telomere length using telomere HPA methods was not associated with endothelial function. Aged and hypertensive changes of the brain (ARWMCs) appear as hyperintense foci on T2-weighted MRI (Pantoni and Garcia, 1997). Patients with severe ARWMCs exhibit an elevated long-term risk of stroke recurrence and unfavorable stroke outcomes (Palumbo et al., 2007, Neumann-Haefelin et al., 2006). The pathogenesis of ARWMCs is thought to be associated with endothelial dysfunction. In addition, shorter leukocyte telomere length measured using quantitative real-time polymerase chain reaction (PCR) was associated with the severity of ARWMCs in non-demented community-based subjects after adjustment for age, sex and some confounders (Wikgren et al., 2014). In our study, the total telomere length of leukocytes, as measured using telomere HPA methods, was slightly associated with the severity of ARWMCs in univariate analysis. However, the association between total telomere length and the severity of ARWMCs was not significant on multivariate analysis. The inconsistencies between our present and these previous results might have been due to differences in baseline characteristics, or in methods used to measure telomere length and evaluate the severity of ARWMCs.

Compared with previous studies, the most interesting factor of our study is its evaluation of telomere G-tail length. Associations between telomere G-tail length and total telomere length have been studied in human endothelial cells from umbilical cord veins (HUVEC) (Anno et al., 2007). Here, we found that telomere G-tail length was positively correlated with total telomere length in humans. However, it remained unclear whether telomere G-tail length was a useful surrogate marker of aging and cardiovascular risk factors in humans. Our study showed that the distribution of associations between telomere G-tail length and total telomere length were significantly different from that in control subjects. This different distribution might indicate that telomere G-tail length is more strongly influenced by cardiovascular risk factors than is total telomere length. Recently, Hirashio et al. reported that shorter telomere G-tail length was associated with future cardiovascular risk in hemodialysis patients, but that total telomere length did not predict cardiovascular events (Hirashio et al., 2014). In addition, our study demonstrated that telomere G-tail length is significantly correlated with endothelial function and the severity of ARWMCs after adjustment for age, sex and baseline characteristics, although the association between total telomere length and these factors was not significant. This result supports the hypothesis that the telomere G-tail is a more important factor in chromosome maintenance than total telomere length in humans.

This study has several limitations. First, our sample size was small, and the causal relationship with leukocyte telomere G-tail length, obtained from cross-sectional data, might be relatively weak. In addition, cross-sectional studies on the measurement of telomere length are unable to evaluate the direct effect of time-dependent changes associated with cardiovascular risk. A better understanding of whether the rate of telomere G-tail length shortening differs from cardiovascular risk and endothelial dysfunction will require longitudinal studies in a larger number of patients. Second, the telomere HPA method is not commonly used, and TRF analysis by Southern blot remains the “gold standard” for measuring telomere length. Some conventional methods, such as quantitative real-time PCR, may be more suitable to detect the relationship between total telomere length and age-related risk factors, as mentioned above (Wikgren et al., 2014). However, telomere HPA does not require an electrophoresis or amplification step and enables a wide range of quantitative analysis (Tahara et al., 2005). In addition, telomere HPA results are consistent with TRF analysis using Southern blot in vitro and in vivo, as previously described (Anno et al., 2007). Accordingly, we consider that telomere HPA is suitable for the measurement of telomere length in clinical samples. Regarding telomere G-tail length, as described previously (Tahara et al., 2005), an appropriate technique to accurately measure G-tail length had not been established. To solve this issue, we previously developed a telomere G-tail HPA method (Tahara et al., 2005) which has the advantages of simple use, accuracy, and high sensitivity for G-tails in vitro. Additionally, recently we developed high-throughput automated machine for G-tail telomere HPA that is applicable for clinical use (Hirashio et al., 2014). Telomere G-tail HPA methods might therefore be more suitable for clinical use than other G-tail length assays. In fact, the telomere G-tail HPA method has already been used to measure total telomere length and G-tail length in practical clinical applications on a commercial clinical basis (MiRTeL Co. LTD, Hiroshima, Japan).

In conclusion, telomere G-tail length might be a useful marker for the severity of ARWMCs and endothelial function as evaluated by FMD. A conclusive determination of the value of telomere G-tail length in predicting the incidence of cerebral and cardiovascular events in general populations awaits larger prospective studies.

## Sources of Funding

This study was supported in part by research grants from the Smoking Research Foundation, the Tsuchiya Foundation, the Japan Science and Technology Agency, the Japan Heart Foundation, Scientific Support Programs for Cancer Research Grant-in-Aid for Scientific Research on Innovative Areas from the Ministry of Education, Culture, Sports, Science and Technology of Japan, and JSPS KAKENHI Grants-in-Aid for Scientific Research (B) (Generative Research Fields).

## Role of the Funding Sources

Research grants were received from the Smoking Research Foundation, the Tsuchiya Foundation, the Japan Science and Technology Agency (AS242Z02592P), the Japan Heart Foundation, the Scientific Support Programs for Cancer Research Grant-in-Aid for Scientific Research on Innovative Areas (221S0001) from the Ministry of Education, Culture, Sports, Science and Technology of Japan, and JSPS KAKENHI Grants-in-Aid for Scientific Research (B) (Generative Research Fields, Grant number 26310106). These grants supported part of the laboratory work and writing of this report.

## Conflicts of Interest/Disclosures

HT is a founder and the board director of MiRTeL Co. LTD. and owns stock in MiRTeL Co. LTD.

## Author Contributions

Study concept and design by TN, NH and TT; clinical data acquisition by TN, NH and SA; laboratory data acquisition by TN, KA, AS, TH and HT; data analysis and interpretation by TN, NH, TT, KA, HM, HT and MM; manuscript drafting by TN, NH, KA and HT; critical revision of the manuscript for important intellectual content by all authors; study supervision by MM.

## Figures and Tables

**Table 1:** Baseline characteristics.

	n = 102
Age (years)	70.1 ± 9.2
Male	69 (67.6)
Body mass index (kg/m^2^)	22.8 ± 3.0
Smoker	8 (7.8)
Hypertension	72 (70.6)
Diabetes mellitus	30 (29.4)
Dyslipidemia	63 (61.8)
Atrial fibrillation	15 (14.7)
Renal dysfunction	34 (33.3)
Systolic blood pressure (mm Hg)	132.0 ± 20.2
Diastolic blood pressure (mm Hg)	77.8 ± 12.8
History of stroke	72 (70.6)
History of coronary artery disease	10 (9.8)
Framingham risk scores	16 (13–18.3)
Physiological findings	
FMD (%)	4.25 ± 2.15
NMD (%)	13.30 ± 5.04
MRI findings (ARWMCs)	
Fazekas rating scores	1 (1–2)
Scheltens rating scores	13 (7–18.3)
Laboratory findings	
White blood cell (10^3^/μl)	5.78 ± 1.93
HbA1c (%)	5.95 ± 0.75
FBG (mg/dl)	111.8 ± 24.3
LDL cholesterol (mg/dl)	109.4 ± 26.3
HDL cholesterol (mg/dl)	63.9 ± 21.3
TG (mg/dl)	105.6 ± 46.1
eGFR (ml/min/1.73 m^2^)	66.9 ± 17.5
Telomere G-tail length (RLU/μg DNA)	13653.0 ± 2787.4
Total telomere length (RLU/μg DNA)	176698.3 ± 20308.0

The data are presented as the means ± SD for age, body mass index, systolic blood pressure, diastolic blood pressure, FMD, NMD and laboratory findings; the medians (interquartile ranges) for Framingham risk scores, Fazekas rating scale and Scheltens rating scale; and the number (%) of patients.

FMD, flow-mediated dilation; NMD, nitroglycerin-mediated dilation; MRI, magnetic resonance imaging; ARWMCs, age-related white matter changes; FBG, fasting blood glucose; LDL, low-density lipoprotein; HDL, high-density lipoprotein; TG, triglycerides; eGFR, estimated glomerular filtration rate; RLU, relative light unit; DNA, deoxyribonucleic acid.

**Table 2:** Patient characteristics according to telomere G-tail length tertiles.

	Telomere G tail lengths			P
Lowest tertile (n = 34)	Middle tertile (n = 34)	Highest tertile (n = 34)
Age	72.6 ± 8.0	71.1 ± 7.7	66.6 ± 10.8	0.018
Male	22 (64.7)	22 (64.7)	25 (73.5)	0.668
Body mass index (kg/m^2^)	22.8 ± 3.6	22.7 ± 3.0	22.9 ± 2.4	0.947
Smoker	2 (5.9)	4 (11.8)	2 (5.9)	0.581
Hypertension	24 (70.6)	27 (79.4)	21 (61.8)	0.279
Diabetes mellitus	12 (35.3)	13 (38.2)	5 (14.7)	0.068
Dyslipidemia	22 (64.7)	23 (67.7)	18 (52.9)	0.418
Atrial fibrillation	3 (8.8)	7 (20.6)	5 (14.7)	0.391
Renal dysfunction	14 (41.2)	9 (26.5)	11 (32.4)	0.433
Systolic blood pressure (mm Hg)	132.1 ± 21.5	136.2 ± 20.9	127.9 ± 17.6	0.237
Diastolic blood pressure (mm Hg)	75.2 ± 9.6	78.7 ± 16.4	79.5 ± 11.3	0.343
History of stroke	24 (70.6)	25 (73.5)	23 (67.7)	0.868
History of coronary artery disease	4 (11.8)	2 (5.9)	4 (11.8)	0.642
Framingham risk scores	17 (11.8–20)	17 (15.5–18)	14 (11.8–16.3)	0.012
Physiological findings				
FMD (%)	3.5 ± 1.5	4.4 ± 2.4	4.8 ± 2.4	0.044
NMD (%)	12.1 ± 4.0	14.1 ± 4.8	13.7 ± 6.0	0.224
MRI findings				
Fazekas rating scores	2 (1–2)	1 (1–2)	1 (0–2)	0.357
Scheltens rating scores	16 (9.8–21.3)	12 (7.8–17.3)	10 (5–15.8)	0.036

The data are presented as the means ± SD for age, body mass index, systolic blood pressure, diastolic blood pressure, FMD and NMD; the medians (interquartile ranges) for Fazekas rating scores and Scheltens rating scores; and the number (%) of patients.

FMD, flow-mediated dilation; NMD, nitroglycerin-mediated dilation; RLU, relative light unit; DNA, deoxyribonucleic acid.

**Table 3:** Associations between vascular risk factors, laboratory findings and flow-mediated dilation (FMD).

Flow-mediated dilation				
	Spearman's correlation		Multiple linear regression	
ρ	P	β coefficient	P
Age	− 0.266	0.007	− 0.229	0.016
Male	− 0.309	0.002	− 0.233	0.010
Body mass index (kg/m^2^)	− 0.255	0.010	–	–
Smoker	− 0.040	0.692	–	–
Hypertension	− 0.305	0.002	− 0.155	0.099
Diabetes mellitus	− 0.277	0.005	–	–
Dyslipidemia	− 0.093	0.351	–	–
Atrial fibrillation	0.056	0.576	–	–
Renal dysfunction	− 0.208	0.036	–	–
Systolic blood pressure (mm Hg)	− 0.079	0.429	–	–
Diastolic blood pressure (mm Hg)	0.082	0.415	–	–
History of stroke	− 0.106	0.287	–	–
History of coronary artery disease	− 0.095	0.344	–	–
Laboratory findings				
White blood cells (10^3^/μl)	− 0.012	0.902	–	–
HbA1c (%)	− 0.187	0.060	–	–
FBG (mg/dl)	− 0.161	0.106	–	–
LDL cholesterol (mg/dl)	− 0.045	0.650	–	–
HDL cholesterol (mg/dl)	0.254	0.010	–	–
TG (mg/dl)	− 0.280	0.004	− 0.220	0.013
eGFR (ml/min/1.73 m^2^)	0.199	0.045	–	–
hs-CRP, log (ng/ml)	− 0.224	0.024	–	–
Telomere G-tail length (RLU/μg DNA)	0.214	0.031	0.205	0.022

β coefficient means standardized partial regression coefficient.

FBG, fasting blood glucose; LDL, low-density lipoprotein; HDL, high-density lipoprotein; TG, triglycerides; eGFR, estimated glomerular filtration rate; RLU, relative light unit; DNA, deoxyribonucleic acid.

**Table 4:** Associations between vascular risk factors, laboratory findings and age-related white matter changes.

Age-related white matter changes				
	Spearman's correlation		Multiple linear regression	
ρ	P	β coefficient	P
Age	0.376	< 0.001	0.372	< 0.001
Male	0.041	0.683	–	–
Body mass index (kg/m^2^)	0.102	0.309	–	–
Smoker	0.025	0.805	–	–
Hypertension	0.263	0.008	–	–
Diabetes mellitus	0.192	0.053	–	–
Dyslipidemia	− 0.020	0.843	–	–
Atrial fibrillation	− 0.134	0.180	–	–
Renal dysfunction	0.082	0.414	–	–
Systolic blood pressure (mm Hg)	0.164	0.100	–	–
Diastolic blood pressure (mm Hg)	0.004	0.971	–	–
History of stroke	0.022	0.824	–	–
History of coronary artery disease	0.101	0.313	–	–
Laboratory findings				
White blood cells (10^3^/μl)	− 0.052	0.603	–	–
HbA1c (%)	0.215	0.030	–	–
FBG (mg/dl)	0.203	0.041	–	–
LDL cholesterol (mg/dl)	− 0.039	0.701	–	–
HDL cholesterol (mg/dl)	− 0.069	0.490	–	–
TG (mg/dl)	0.063	0.532	–	–
eGFR (ml/min/1.73 m^2^)	− 0.155	0.121	–	–
hs-CRP, log (ng/ml)	0.043	0.670	–	–
Telomere G-tail length (RLU/μg DNA)	− 0.309	0.002	− 0.200	0.033

β coefficient means standardized partial regression coefficient.

FBG, fasting blood glucose; LDL, low-density lipoprotein; HDL, high-density lipoprotein; TG, triglycerides; eGFR, estimated glomerular filtration rate; RLU, relative light unit; DNA, deoxyribonucleic acid.
