# Supplementary material for: Telomere G-tail Length is a Promising Biomarker Related to White Matter Lesions and Endothelial Dysfunction in Patients With Cardiovascular Risk: A Cross-sectional Study
Source: eBioMedicine. 2015 May 30;2(8):960–7. doi: 10.1016/j.ebiom.2015.05.025 (PMC4563121; doi:10.1016/j.ebiom.2015.05.025)
Supplement: Supplementary file 1 — Supplementary tables. [file mmc1.doc]

**Supplement Table I.**

Correlation of telomere G-tail lengths or total telomere lengths with laboratory findings

|  | **G-tail** | | **Total telomere** | |
| --- | --- | --- | --- | --- |
| **Laboratory findings** | ρ | P | ρ | P |
| White blood cells (10^3^/µl) | 0.166 | 0.096 | 0.093 | 0.355 |
| HbA1c (%) | -0.161 | 0.105 | -0.175 | 0.078 |
| FBG (mg/dl) | 0.064 | 0.524 | -0.090 | 0.367 |
| LDL cholesterol (mg/dl) | 0.097 | 0.334 | -0.002 | 0.985 |
| HDL cholesterol (mg/dl) | -0.001 | 0.950 | 0.055 | 0.581 |
| TG (mg/dl) | -0.048 | 0.629 | -0.097 | 0.335 |
| eGFR (ml/min/1.73 m^2^) | 0.166 | 0.096 | 0.099 | 0.322 |
| hs-CRP, log (ng/ml) | 0.135 | 0.176 | -0.104 | 0.297 |

FBG, fasting blood glucose; LDL, low-density lipoprotein; HDL, high-density lipoprotein; TG, triglycerides; eGFR, estimated glomerular filtration rate; hs-CRP, high-sensitivity C-reactive protein

**Supplement Table II.**

Comparison of characteristics between the patients included in this study and control subjects

|  | Patients included in the study  n=102 | Control subjects  n=102 | **P** |
| --- | --- | --- | --- |
| Age, yr | 70.1±9.2 | 68.8±6.1 | 0.249 |
| Male | 69 (67.7) | 64 (62.3) | 0.557 |
| Telomere G-tail length (RLU/µg DNA) | 13653.0±2787.4 | 22504.9±3249.1 | <0.001 |
| Total telomere length (RLU/µg DNA) | 176698.3±20308.0 | 181305.0±22234.7 | 0.124 |

The data are presented as the means±SD for age, telomere G-tail length and total telomere length and the number (%) of male patients.

RLU, relative light unit; DNA, deoxyribonucleic acid

**Supplement Table III.** Patient characteristics according to total telomere length tertile

|  | **Total telomere lengths** | | | **P** |
| --- | --- | --- | --- | --- |
|  | Lowest tertile  (n=34) | Middle tertile  (n=34) | Highest tertile  (n=34) |  |
| Age | 74.5±6.5 | 69.2±10.1 | 66.6±9.1 | 0.001 |
| Male | 23 (67.7) | 23 (67.7) | 23 (67.7) | 1.000 |
| Body mass index (kg/m^2^) | 22.5±3.2 | 22.9±2.9 | 23.0±2.9 | 0.730 |
| Smoker | 3 (8.8) | 4 (11.8) | 1 (2.9) | 0.387 |
| Hypertension | 27 (79.4) | 20 (58.8) | 25 (73.5) | 0.159 |
| Diabetes mellitus | 13 (38.2) | 9 (26.5) | 8 (23.5) | 0.371 |
| Dyslipidemia | 23 (67.7) | 17 (50.0) | 23 (67.7) | 0.224 |
| Atrial fibrillation | 4 (11.8) | 7 (20.6) | 4 (11.8) | 0.495 |
| Renal dysfunction | 13 (38.2) | 11 (32.4) | 10 (29.4) | 0.734 |
| Systolic blood pressure (mmHg) | 136.0±23.6 | 129.6±16.5 | 130.5±19.8 | 0.376 |
| Diastolic blood pressure (mmHg) | 78.0±13.1 | 78.8±13.1 | 76.6±12.4 | 0.781 |
| History of stroke | 25 (73.5) | 24 (70.6) | 23 (67.7) | 0.868 |
| History of coronary artery disease | 2 (5.9) | 5 (14.7) | 3 (8.8) | 0.460 |
| Framingham risk scores | 17 (14-21) | 16.5 (12.8-19) | 14.5 (11.8-16.3) | 0.026 |
| **Physiological findings** |  |  |  |  |
| FMD (%) | 3.6±1.5 | 4.6±2.3 | 4.6±2.5 | 0.085 |
| NMD (%) | 12.3±3.8 | 14.1±5.6 | 13.5±5.6 | 0.321 |
| **MRI findings** |  |  |  |  |
| Fazekas rating scores | 2 (1-2) | 1 (0-2) | 1 (1-2) | 0.068 |
| Scheltens rating scores | 17 (10.8-21.3) | 11.5 (5-17.3) | 12 (7-17) | 0.022 |

The data are presented as the means±SD for age, body mass index, systolic blood pressure, diastolic blood pressure, FMD and NMD; the medians (interquartile ranges) for Fazekas rating scores and Scheltens rating scores; and the number (%) of patients.

FMD, flow-mediated dilation; NMD, nitroglycerin-mediated dilation; RLU, relative light unit; DNA, deoxyribonucleic acid
